# Supplementary material for: Time Series Data Provide Insights into the Evolution and Abundance of One of the Most Abundant Viruses in the Marine Virosphere: The Uncultured Pelagiphages vSAG 37-F6
Source: Viruses. 2024 Oct 24;16(11):1669. doi: 10.3390/v16111669 (PMC11598899; doi:10.3390/v16111669)
Supplement: Supplementary file 1 [file viruses-16-01669-s001.zip › viruses-3215587-supplementary.pdf]

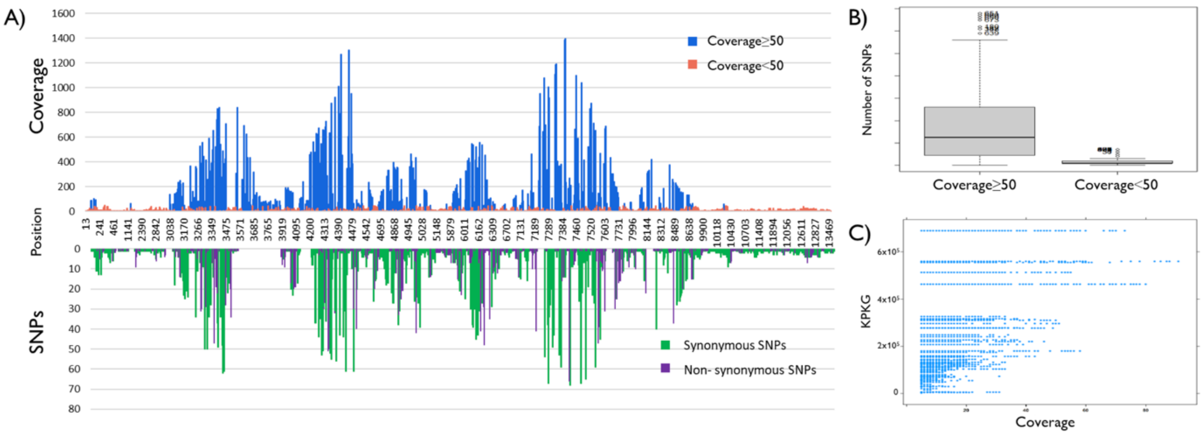

Supplementary Figure S1. Abundance and distribution of SNPs in the 37-F6 genome based on 7-year temporal sampling. A) Total coverage at each position with colors indicating coverage over 50x (blue) or falling below (red). Additionally, the total number of SNPs at each position is shown, including both synonymous (green) and non-synonymous (purple) mutations. B) The number of SNPs exhibits a direct correlation with position coverage, depending on whether the coverage surpasses (R^2=0.67) or falls below (R^2=0.41) the 50x threshold. C) Coverage show no correlation with sample abundance (KPKG) (R^2=0.17), probing that the elevated number of SNPs in certain genome regions is due to the high coexisting microdiversity from the virus as most of the SNPs are present in position with coverage above 50x.

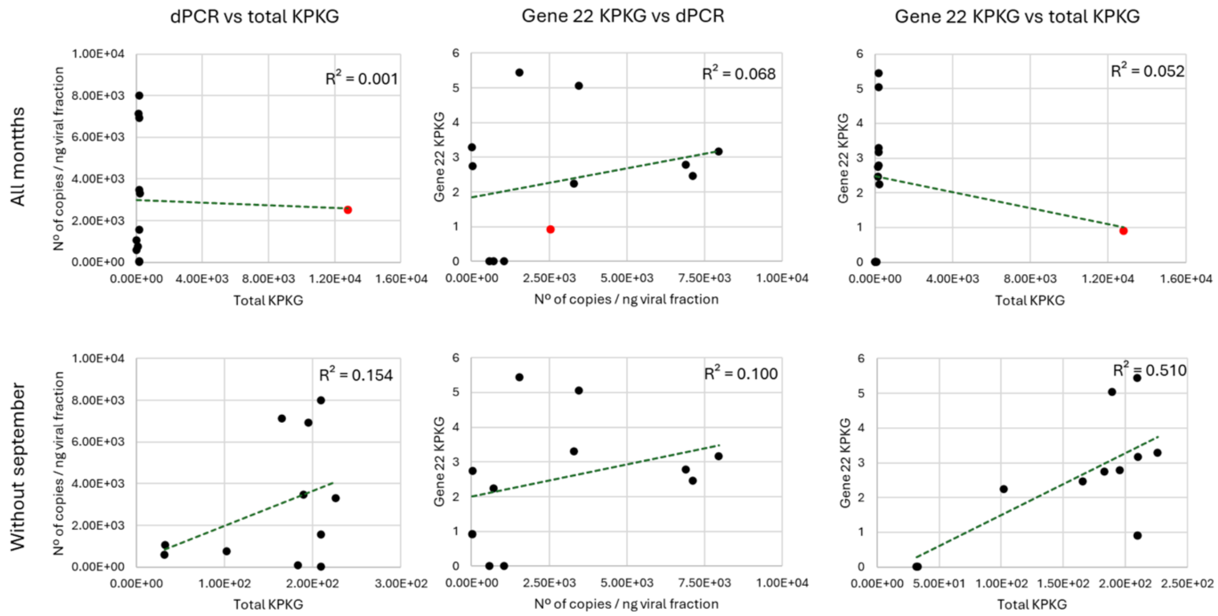

Supplementary Figure S2. Relationship between two methods for quantifying viral abundance: dPCR and KPKG. For the KPKG method, two values are considered: total KPKG and KPKG calculated using only the gene targeted by the dPCR primers (gene 22), to assess potential differences. The correlation between these parameters was examined across all months, excluding September as it is an outlier, to investigate potential impacts of its exclusion.
